# Spiritual Rappers

**Published:** 1853-05

**Authors:** 


*Spiritual Rappers.* — We meet frequently with notices of insanity produced by belief in spiritual rapping. In the April No. of the New York Medical Times, it is stated that during the past year eighteen persons were admitted into the Indiana Lunatic Asylum, whose insanity was ascribed to this belief. If the delusion continue to prevail, the subject may perhaps assume sufficient importance, by and by, not to be beneath the notice of even so dignified a body as the American Medical Association!

